# Sex-specific clinical profiles in patients with acute ischemic stroke and chronic kidney disease

**DOI:** 10.3389/fneur.2026.1959768

**Published:** 2026-10-02

**Authors:** Sean Holden-Kapshuck, Danny Pham, Grey Holler, Georgia Roper, Samuel Nathaniel, Austin Dang, Brandon Phillips, Mason Pearce, Richard Goodwin, Thomas I. Nathaniel

**Affiliations:** 1 Department of Biomedical Sciences, University of South Carolina School of Medicine Greenville, Greenville, SC, United States; 2 Department of Biomedical Engineering, University of South Carolina, Columbia, SC, United States

**Keywords:** BP, chronic kidney disease, men, sleep apnea, stroke, women

## Abstract

**Background:**

Chronic kidney disease (CKD) is common among patients with acute ischemic stroke (AIS) and is associated with worse outcomes. Although sex-related differences in stroke are well recognized, it remains unclear whether clinical characteristics associated with sex differ according to CKD status. We characterized sex-associated clinical profiles among patients with AIS with and without documented CKD.

**Methods:**

We conducted a retrospective analysis of the PRISMA Health Stroke Registry. Baseline characteristics were compared between patients with and without documented CKD in the overall cohort and after stratification by sex. Multivariable logistic regression with backward likelihood-ratio selection was used to identify independent clinical characteristics associated with documented CKD in the overall cohort and with male sex within the CKD and non-CKD subgroups. Adjusted odds ratios (ORs) and 95% confidence intervals (CIs) were calculated. Internal stability was assessed using bootstrap resampling.

**Results:**

Among 5,469 patients with AIS, 447 (8.2%) had documented CKD. Independent characteristics associated with CKD included prosthetic heart valve (OR, 3.73), elevated serum creatinine (OR, 2.79), obesity (OR, 2.51), heart failure (OR, 2.21), hypertension (OR, 2.20), African American race (OR, 2.11), and depression (OR, 1.97). In stratified analyses, associations of male sex with sleep apnea, smoking, and diastolic blood pressure ≥80 mmHg were numerically stronger in the CKD group than in the non-CKD group: sleep apnea, OR 4.62 vs. 2.38; smoking, OR 3.19 vs. 1.31; and diastolic blood pressure ≥80 mmHg, OR 2.58 vs. 1.43. However, formal interaction testing did not demonstrate statistically significant effect modification by CKD status. The sex-association model among patients with CKD demonstrated greater apparent discrimination than the corresponding non-CKD model (AUC, 0.785 vs. 0.630).

**Conclusion:**

Patients with AIS demonstrated distinct sex-associated clinical profiles according to CKD status. Although several associations were numerically stronger among patients with CKD, formal interaction testing did not establish statistically significant effect modification. These findings characterize clinical heterogeneity among men and women with established AIS and warrant further investigation to determine whether these profiles have implications for treatment or outcomes.

## 1 Introduction

Acute ischemic stroke (AIS) is the second-leading cause of death and the third-leading cause of disability-adjusted life years worldwide. Although age-standardized rates have declined, the absolute global burden continues to increase with population aging (1, 2). Outcomes after ischemic stroke are influenced not only by infarct characteristics but also by the constellation of comorbid conditions present at presentation. Because patients with AIS constitute a heterogeneous population, identifying subgroups with distinct clinical profiles is important for risk stratification, appropriate allocation of interventions, and understanding outcome variation among patients with the same diagnostic condition.

Chronic kidney disease (CKD) is one of the most clinically consequential comorbidities in patients with AIS. In a meta-analysis of 263,633 patients with anterior-circulation AIS who received reperfusion therapy, the pooled prevalence of CKD ranged from 30% to 56% among patients treated with intravenous thrombolysis and from 16% to 42% among those treated with endovascular thrombectomy (3). In the same analysis, CKD was associated with greater odds of an unfavorable functional outcome at 90 days after both intravenous thrombolysis and endovascular thrombectomy (3). CKD has also been independently associated with poorer functional recovery, increased hemorrhagic risk, and higher mortality (4). Lower estimated glomerular filtration rate (eGFR) is additionally associated with greater National Institutes of Health Stroke Scale (NIHSS) scores and poorer functional status at baseline and 3 months after stroke (5).

The association between renal impairment and cerebrovascular injury may be explained in part by the shared vascular vulnerability of the kidneys and brain. Both are highly perfused organs that rely on precise local autoregulation to maintain tissue integrity (6, 7). Consequently, both organs are susceptible to hemodynamic stress and atherosclerotic injury, while shared vascular risk factors—including hypertension, diabetes mellitus, and smoking—may contribute to parallel progression toward a systemic small-vessel disease phenotype (6, 8). Even mild renal dysfunction has been associated with acute and chronic cerebrovascular disease, and renal impairment may increase the risk of cerebrovascular events, including intracerebral hemorrhage (6). Endothelial dysfunction, chronic inflammation, oxidative stress, accelerated vascular calcification, and disturbances in platelet and coagulation function further increase vascular risk in CKD (9, 10). Thus, CKD is characterized by the simultaneous presence of elevated thrombotic risk and increased bleeding susceptibility, related in part to uremic toxin accumulation, platelet dysfunction, anemia, and impaired vascular integrity (9, 11). This combination complicates decisions regarding thrombolysis, antiplatelet therapy, and anticoagulation and is further compounded by the exclusion of patients with advanced renal disease from many major randomized antithrombotic trials (10). CKD may therefore represent more than an additional comorbidity; it may also identify a distinct clinical context in which sex-associated characteristics of AIS differ.

Sex-related differences in ischemic stroke are well established, independent of renal status. Women are generally older at presentation and have higher prevalences of hypertension and atrial fibrillation, whereas men may have greater stroke risk associated with hyperlipidemia and other vascular risk factors (12–14). These differences may also extend to treatment and outcomes. Among patients with atrial fibrillation and ischemic stroke, women are less likely than men to receive anticoagulation at discharge (15). In addition, the higher post-stroke mortality observed among women compared with men has been attributed in part to differences in pre-stroke functional status, age, and stroke severity (15, 16).

Sex-related differences also influence the diagnosis and interpretation of CKD. Women generally have lower serum creatinine concentrations than men at comparable measured levels of glomerular filtration, primarily because of differences in muscle mass and creatinine production (17, 18). Differences have also been reported in nephrology referral, CKD monitoring, and CKD progression between women and men (17–20). Furthermore, sex-specific analyses of the CKD–stroke relationship suggest potential heterogeneity, with CKD reported to be associated more strongly with hemorrhagic stroke among men and ischemic stroke among women (21). These findings suggest that sex-associated clinical characteristics among patients with AIS should not be assumed to be uniform across CKD strata. To our knowledge, no prior study has directly compared the adjusted clinical characteristics associated with male vs. female sex among patients with AIS with CKD and those without CKD.

Although sex differences in AIS and CKD have been characterized independently, less is known about whether the multivariable clinical profile distinguishing men from women with established AIS varies according to renal status. The present study addresses this gap by comparing sex-associated demographic, cardiovascular, metabolic, and clinical characteristics within CKD and non-CKD strata of the same AIS cohort. We also formally evaluated CKD-dependent heterogeneity for selected shared associations, thereby distinguishing differences in the composition of CKD-stratified profiles from statistical effect modification. We sought to identify factors independently associated with male vs. female sex within CKD and non-CKD subgroups and to determine whether CKD status modified selected sex-associated clinical characteristics among patients with AIS.

## 2 Methods

### 2.1 Study population

This observational study used 6 years of retrospective data from the PRISMA Health Stroke Registry. The study was approved by the PRISMA Health Institutional Review Board (approval number Pro00046787). All data were fully anonymized before access, and informed consent was waived in accordance with applicable national legislation and institutional requirements. The analytic cohort included 5,469 unique patients with acute ischemic stroke (AIS). Each patient contributed only one index AIS encounter; therefore, multiple encounters from the same individual were not analyzed as separate observations. A history of previous or recurrent stroke was retained as a clinical characteristic. Because each patient contributed one observation, the analyses did not require adjustment for within-patient clustering.

### 2.2 Data collection

Ischemic stroke was identified through neurological assessment and brain imaging with computed tomography (CT) or magnetic resonance imaging (MRI). MRI, magnetic resonance angiography, CT, or CT angiography was used to confirm ischemic stroke and exclude intracerebral or subarachnoid hemorrhage. Eligible events were coded as ICD-9 434 (occlusion of cerebral arteries) or ICD-10 I63 (cerebral infarction), including both initial and recurrent events. Encounters coded solely as transient ischemic attack (TIA; ICD-9 435 or ICD-10 G45) were excluded because advances in neuroimaging have resulted in many events previously classified as TIA being recognized as minor ischemic strokes. Patients with suspected or confirmed hemorrhagic stroke or missing variables required for the adjusted analyses were also excluded.

Patients were categorized into two mutually exclusive groups according to the presence or absence of documented chronic kidney disease (CKD). CKD status was based on the clinical diagnosis recorded in the PRISMA Health Stroke Registry. This classification represents documented clinical CKD rather than renal dysfunction independently adjudicated according to contemporary Kidney Disease: Improving Global Outcomes criteria. The analytic dataset did not include longitudinal estimated glomerular filtration rate (eGFR) measurements, albuminuria, CKD duration or stage, or dialysis status sufficient for such adjudication.

Baseline characteristics included demographic factors, comorbidities, medication use, vital signs, laboratory measurements, BMI, and functional status. Demographic variables included sex, age, race, and ethnicity. Clinical history variables included atrial fibrillation, coronary artery disease, carotid artery stenosis, depression, diabetes, dyslipidemia, heart failure, hypertension, obesity, previous stroke, prosthetic heart valve, peripheral vascular disease, sleep apnea, and smoking. Medication variables included antihypertensive, lipid-lowering, antidiabetic, antidepressant, antiplatelet, and anticoagulant therapy (See Table 1).

**Table 1:** Demographic and clinical characteristics of patients with acute ischemic stroke, with and without documented chronic kidney disease (CKD).

Characteristic	CKD	No CKD	*P*-value
Number of patients	447	5,022	
**Demographics**			
Age Group: No. (%)			0.022^*^a
< 50	36 (8.1)	622 (12.4)	
50–59	75 (16.8)	921 (18.3)	
60–69	102 (22.8)	1,197 (23.8)	
70–79	113 (25.3)	1,118 (22.3)	
≥80	121 (27.1)	1,164 (23.2)	
Age, years	69.48 ± 13.769	67.05 ± 14.801	< 0.001^*^b
Race: No. (%)			< 0.001^*^a
Caucasians	287 (64.2)	4,001 (79.7)	
African Americans	153 (34.2)	849 (16.9)	
Other	7 (1.6)	172 (3.4)	
Hispanic ethnicity: No. (%)	7 (1.6)	78 (1.6)	0.983^a^
BMI, kg/m^2^	28.83 ± 7.405	28.30 ± 6.937	0.125^b^
**Medical history: No. (%)**			
Atrial fibrillation	103 (23.0)	821 (16.3)	< 0.001^*^a
Coronary artery disease	191 (42.7)	1,470 (29.3)	< 0.001^*^a
Carotid artery stenosis	42 (9.4)	292 (5.8)	0.002^*^a
Depression	101 (22.6)	620 (12.3)	< 0.001^*^a
Diabetes	247 (55.3)	1,688 (33.6)	< 0.001^*^a
Drug or alcohol use	32 (7.2)	305 (6.1)	0.360^a^
Dyslipidemia	290 (64.9)	2,465 (49.1)	< 0.001^*^a
Heart failure	110 (24.6)	480 (9.6)	< 0.001^*^a
Hypertension	412 (92.2)	3,894 (77.5)	< 0.001^*^a
Migraine	9 (2.0)	125 (2.5)	0.533^a^
Obesity	290 (64.9)	2,021 (40.2)	< 0.001^*^a
Previous stroke	149 (33.3)	1,275 (25.4)	< 0.001^*^a
Previous TIA (>24 h)	42 (9.4)	435 (8.7)	0.598^a^
Prosthetic heart valve	11 (2.5)	51 (1.0)	0.006^*^a
Peripheral vascular disease	66 (14.8)	334 (6.7)	< 0.001^*^a
Sleep apnea	29 (6.5)	141 (2.8)	< 0.001^*^a
Smoker	93 (20.8)	1,393 (27.7)	0.002^*^a
**Medication history: No. (%)**			
HTN medication	371 (83.0)	3,423 (68.2)	< 0.001^*^a
Cholesterol reducer	262 (58.6)	2,166 (43.1)	< 0.001^*^a
Diabetic medication	179 (40.0)	1,316 (26.2)	< 0.001^*^a
Antidepressant	99 (22.1)	612 (12.2)	< 0.001^*^a
Antiplatelet prior	239 (53.5)	2,209 (44.0)	< 0.001^*^a
Anticoagulant prior	51 (11.4)	412 (8.2)	0.020^*^a
Initial NIHSS score	8.36 ± 8.594	8.28 ± 8.214	0.857^b^
**Lab values: Mean** ±**SD**			
Total cholesterol, mg/dL	164.36 ± 53.059	172.47 ± 51.667	0.004^*^b
Triglycerides, mg/dL	145.61 ± 106.267	139.14 ± 105.063	0.259^b^
HDL, mg/dL	38.81 ± 13.355	42.03 ± 13.853	< 0.001^*^b
LDL, mg/dL	99.25 ± 44.569	105.07 ± 40.967	0.009^*^b
Lipids	6.70 ± 1.893	6.52 ± 2.609	0.200^b^
Blood glucose, mg/dL	165.32 ± 104.233	145.68 ± 78.441	< 0.001^*^b
Serum creatinine, mg/dL	2.79 ± 2.297	1.16 ± 0.895	< 0.001^*^b
INR	1.19 ± 0.493	1.14 ± 0.496	0.090^b^
**Vital signs: Mean** ±**SD**			
Heart rate, bpm	83.35 ± 20.199	81.88 ± 18.370	0.140^b^
Blood pressure, systolic, mmHg	151.20 ± 31.569	151.88 ± 29.107	0.640^b^
Blood pressure, diastolic, mmHg	79.53 ± 21.049	82.70 ± 18.920	0.002^*^b
**Ambulation status prior to event: No. (%)**			
Ambulate independently	367 (82.1)	4,520 (90.0)	< 0.001^*^a
Ambulate with assistance	21 (4.7)	182 (3.6)	0.250^a^
Unable to ambulate	26 (5.8)	187 (3.7)	0.028^*^a
Not documented	33 (7.4)	132 (2.6)	< 0.001^*^a
rtPA received: No. (%)	79 (17.7)	1,248 (24.9)	< 0.001^*^a
First care received: emergency department, No. (%)	74 (16.6)	1,050 (20.9)	0.024^*^a
Diastolic blood pressure ≥ 80 mmHg: No. (%)	208 (46.5)	2,680 (53.4)	0.005^*^a

Continuous variables are presented as mean ± standard deviation (SD), and categorical variables as *n* (%). Categorical variables were compared using Pearson's chi-square test and continuous variables using Student's *t* test.

a Pearson's chi-square test.

b Student's t-test.

* *P*-value < 0.05.

Laboratory and physiologic variables included total cholesterol, triglycerides, HDL cholesterol, LDL cholesterol, blood glucose, serum creatinine, international normalized ratio (INR), heart rate, and diastolic blood pressure. BMI was recorded and classified according to World Health Organization guidelines. Ambulation was coded on a 0–3 scale: 0, undocumented ambulation ability; 1, unable to ambulate; 2, able to ambulate with assistance; and 3, able to ambulate independently.

### 2.3 Statistical analysis

Analyses were performed using SPSS version 29.0 for Windows (IBM Corp., Armonk, NY, USA). Continuous variables were summarized as mean ± standard deviation (SD) and compared using Student's *t* test. Categorical variables were summarized as counts and percentages and compared using Pearson's chi-square test. Comparisons between patients with and without CKD were performed in the overall cohort and repeated separately among men and women to assess whether CKD-associated differences were consistent across sex strata.

Multivariable logistic regression was used to evaluate factors associated with documented CKD in the overall AIS cohort and factors associated with sex within the CKD and non-CKD strata. Candidate variables were selected from the corresponding univariate analyses using a screening threshold of *P* < 0.20. This threshold was selected to reduce premature exclusion of potentially relevant covariates whose conditional associations might become apparent after adjustment. Candidate variables were then evaluated using backward likelihood-ratio selection, with *P* < 0.05 defining statistical significance in the final models.

The CKD-stratified sex models were fitted separately rather than as a single pooled model because the primary objective was to characterize sex-associated clinical profiles within each renal-status subgroup and to permit the model structure to vary between strata. In these models, male sex was coded as the outcome (male = 1; female = 0). Accordingly, an OR >1 indicated an independent association with male sex, whereas an OR < 1 indicated an association with female sex. These models characterized sex-associated clinical profiles among patients with established AIS and were not designed to predict AIS occurrence or future stroke risk. Reference categories are specified in the corresponding table legends.

#### 2.3.1 Missing data and predictor selection

Missing data was handled using complete-case analysis. For each multivariable model, observations missing one or more variables required for that model were excluded from the corresponding analysis; no multiple imputation was performed. Consequently, the effective sample size could vary among models. Given the smaller CKD subgroup, the number of complete observations contributing to the CKD-stratified model was specifically examined and is reported with the corresponding results.

Potential conceptual redundancy and multicollinearity were assessed before multivariable modeling. Continuous variables were not entered simultaneously with categorical variables directly derived from the same measure. Thus, continuous age was modeled without concurrent age-category indicators, and continuous BMI was modeled without simultaneous inclusion of obesity status when both represented the same underlying construct. Age categories were evaluated separately where applicable.

Lipid-related variables, including dyslipidemia, LDL cholesterol, HDL cholesterol, and total cholesterol, were assessed for correlation and conceptual overlap. When substantial redundancy was identified, the clinically most interpretable measure was prioritized to avoid unnecessary duplication. Multicollinearity was evaluated using variance inflation factors (VIFs) and tolerance statistics. A VIF >5 and/or tolerance < 0.20 was considered potentially problematic, prompting further assessment of redundancy before inclusion in the final model. This approach was intended to improve coefficient interpretability, reduce model complexity, and limit instability, particularly in the smaller CKD subgroup.

Within the CKD-stratified sex model, three pre-event ambulation variables—independent ambulation, ambulation with assistance, and inability to ambulate—exhibited complete separation and were excluded from the final reported model. Where transformation was required, the fifth root was applied; transformed variables are identified by a caret in the corresponding table and figure legends. Variables with low prevalence or sparse event counts in the CKD subgroup were interpreted cautiously because of the potential for unstable or inflated estimates.

#### 2.3.2 Internal validation and sensitivity analysis

Because data-driven variable selection can produce unstable coefficients and optimistic effect estimates, internal validation was performed using bootstrap resampling. One thousand bootstrap samples were generated. Within each sample, the variable-selection procedure was repeated, and bootstrap ORs, 95% confidence intervals (CIs), and selection frequencies were calculated. Selection frequency was defined as the proportion of bootstrap samples in which a variable was retained in the final model and was used to assess the stability of individual associations.

Firth bias-reduced logistic regression was prespecified as a sensitivity analysis, particularly because conventional maximum-likelihood logistic regression may produce inflated or unstable estimates when predictor frequencies are low or separation occurs. Firth estimates were intended to complement bootstrap validation: bootstrap analysis assessed sampling and variable-selection stability, whereas Firth regression addressed potential small-sample and separation-related bias. Robustness was evaluated by comparing the direction and magnitude of the conventional ORs, bootstrap estimates and selection frequencies, and Firth penalized ORs with profile-likelihood 95% CIs.

#### 2.3.3 Interaction analyses

Formal CKD-dependent heterogeneity was evaluated separately using pooled logistic regression models. Because sex was the dependent variable in the CKD-stratified analyses, the interaction models examined whether the associations between selected clinical characteristics and sex differed according to CKD status. Each pooled model included CKD status, the clinical characteristic of interest, an explicit CKD × clinical-characteristic interaction term, and the relevant adjustment covariates. Interaction terms were prespecified for sleep apnea, smoking, and DBP ≥80 mmHg. Interaction effects were summarized using interaction ORs, 95% CIs, and *P* values for interaction. A *P* interaction < 0.05 was considered evidence of statistically significant effect modification by CKD status. Stratified ORs were retained to describe associations within the CKD and non-CKD subgroups but were not interpreted as evidence of effect modification in the absence of a statistically significant interaction term.

#### 2.3.4 Model performance

Adjusted ORs and 95% CIs were calculated from the corresponding logistic regression coefficients. Statistical significance was defined as *P* < 0.05. Adjusted analyses are presented in forest plots. Confidence intervals entirely below 1 indicate an association with the reference category, whereas intervals entirely above 1 indicate an association with the modeled outcome; intervals crossing 1 indicate no statistically significant association at the 0.05 level. An asterisk denotes *P* < 0.05, and a caret identifies variables transformed using the fifth root. Model performance was assessed using discrimination, calibration, and classification measures. Discrimination was quantified using the area under the receiver operating characteristic curve (AUC), and calibration was assessed using the Hosmer–Lemeshow goodness-of-fit test. Sensitivity, specificity, positive predictive value, negative predictive value, and overall classification accuracy were also calculated at the prespecified classification threshold.

Because CKD was present in only 8.2% of the overall cohort, overall classification accuracy was not considered an adequate standalone measure of performance and was interpreted cautiously. Model performance was therefore assessed primarily using discrimination, calibration, and stability of the estimated associations. The CKD and non-CKD models were developed separately in different patient strata and included different predictor sets. Their AUCs were therefore interpreted as within-sample measures of discrimination and not as direct evidence of differences in biological structure or statistically superior performance between CKD strata.

## 3 Results

### 3.1 Baseline characteristics of AIS patients with and without CKD

A total of 5,469 unique patients with AIS were included, including 447 (8.2%) with documented CKD and 5,022 (91.8%) without CKD. Patients with CKD were older than those without CKD (69.48 ± 13.77 vs. 67.05 ± 14.80 years, *P* < 0.001) and differed in racial composition. The CKD group included a higher proportion of African American patients (34.2% vs. 16.9%) and a lower proportion of White patients (64.2% vs. 79.7%; *P* < 0.001). BMI did not differ significantly between groups (28.83 ± 7.41 vs. 28.30 ± 6.94 kg/m^2^, *P* = 0.125), nor did Hispanic ethnicity (*P* = 0.983).

Patients with CKD had a greater comorbidity burden, including higher prevalences of atrial fibrillation (23.0% vs. 16.3%), coronary artery disease (42.7% vs. 29.3%), carotid artery stenosis (9.4% vs. 5.8%), depression (22.6% vs. 12.3%), diabetes (55.3% vs. 33.6%), dyslipidemia (64.9% vs. 49.1%), heart failure (24.6% vs. 9.6%), hypertension (92.2% vs. 77.5%), obesity (64.9% vs. 40.2%), previous stroke (33.3% vs. 25.4%), prosthetic heart valve (2.5% vs. 1.0%), peripheral vascular disease (14.8% vs. 6.7%), and sleep apnea (6.5% vs. 2.8%) (all *P* < 0.05). They were less likely to smoke (20.8% vs. 27.7%, *P* = 0.002) and more likely to receive antihypertensive, lipid-lowering, antidiabetic, antidepressant, antiplatelet, and anticoagulant therapies (all *P* < 0.05).

Patients with CKD had higher serum creatinine (2.79 ± 2.30 vs. 1.16 ± 0.90 mg/dL, *P* < 0.001) and blood glucose levels (165.32 ± 104.23 vs. 145.68 ± 78.44 mg/dL, *P* < 0.001), but lower total cholesterol, HDL cholesterol, and LDL cholesterol levels (all *P* < 0.05). They were less likely to ambulate independently before the event (82.1% vs. 90.0%, *P* < 0.001) and less likely to receive rtPA (17.7% vs. 24.9%, *P* < 0.001). Initial NIHSS scores did not differ between groups (8.36 ± 8.59 vs. 8.28 ± 8.21, *P* = 0.857).

When stratified by sex (Table 2), CKD-associated differences in comorbidity burden, medication use, and laboratory values were observed in both men and women. Serum creatinine was higher among patients with CKD in both strata: 2.97 ± 2.39 vs. 1.26 ± 0.93 mg/dL in men and 2.59 ± 2.18 vs. 1.06 ± 0.86 mg/dL in women (both *P* < 0.001).

**Table 2:** Demographic and clinical characteristics of patients with acute ischemic stroke, with and without documented chronic kidney disease (CKD), stratified by sex.

Characteristic	Male			Female		
	**CKD**	**No CKD**	***P*** **-value**	**CKD**	**No CKD**	***P*** **-value**
Number of patients	232	2,430		215	2,592	
**Demographics**						
Age group: No. (%)			0.008^*^a			0.061^a^
< 50	20 (8.6)	285 (11.7)		16 (7.4)	337 (13.0)	
50–59	50 (21.6)	553 (22.8)		25 (11.6)	368 (14.2)	
60–69	49 (21.1)	691 (28.4)		53 (24.7)	506 (19.5)	
70–79	64 (27.6)	528 (21.7)		49 (22.8)	590 (22.8)	
≥80	49 (21.1)	373 (15.3)		72 (33.5)	791 (30.5)	
Age, years	67.79 ± 13.776	64.87 ± 13.307	0.002^*^b	71.30 ± 13.559	69.09 ± 15.809	0.025^*^b
Race: No. (%)			< 0.001^*^a			< 0.001^*^a
Caucasians	145 (62.5)	1,945 (80.0)		142 (66.0)	2,056 (79.3)	
African Americans	86 (37.1)	406 (16.7)		67 (31.2)	443 (17.1)	
Other	1 (0.4)	79 (3.3)		6 (2.8)	93 (3.6)	
Hispanic ethnicity	4 (1.7)	34 (1.4)	0.690^a^	3 (1.4)	44 (1.7)	0.740^a^
BMI, kg/m^2^	27.92 ± 6.634	28.06 ± 5.919	0.734^b^	29.82 ± 8.059	28.52 ± 7.766	0.020^*^b
**Medical history: No. (%)**						
Atrial fibrillation	48 (20.7)	333 (13.7)	0.004^*^a	55 (25.6)	488 (18.8)	0.016^*^a
Coronary artery disease	105 (45.3)	830 (34.2)	< 0.001^*^a	86 (40.0)	640 (24.7)	< 0.001^*^a
Carotid artery stenosis	25 (10.8)	166 (6.8)	0.026^*^a	17 (7.9)	126 (4.9)	0.051^a^
Depression	45 (19.4)	226 (9.3)	< 0.001^*^a	56 (26.0)	394 (15.2)	< 0.001^*^a
Diabetes	133 (57.3)	818 (33.7)	< 0.001^*^a	114 (53.0)	870 (33.6)	< 0.001^*^a
Drug or alcohol use	24 (10.3)	232 (9.5)	0.694^a^	8 (3.7)	73 (2.8)	0.446^a^
Dyslipidemia	147 (63.4)	1,217 (50.1)	< 0.001^*^a	143 (66.5)	1,248 (48.1)	< 0.001^*^a
Heart failure	54 (23.3)	191 (7.9)	< 0.001^*^a	56 (26.0)	289 (11.1)	< 0.001^*^a
Hypertension	209 (90.1)	1,847 (76.0)	< 0.001^*^a	203 (94.4)	2,047 (79.0)	< 0.001^*^a
Migraine	3 (1.3)	26 (1.1)	0.754^a^	6 (2.8)	99 (3.8)	0.445^a^
Obesity	146 (62.9)	1,002 (41.2)	< 0.001^*^a	144 (67.0)	1,019 (39.3)	< 0.001^*^a
Previous stroke	81 (34.9)	590 (24.3)	< 0.001^*^a	68 (31.6)	685 (26.4)	0.098^a^
Previous TIA (>24 h)	16 (6.9)	197 (8.1)	0.516^a^	26 (12.1)	238 (9.2)	0.160^a^
Prosthetic heart valve	9 (3.9)	26 (1.1)	< 0.001^*^a	2 (0.9)	25 (1.0)	0.961^a^
Peripheral vascular disease	35 (15.1)	157 (6.5)	< 0.001^*^a	31 (14.4)	177 (6.8)	< 0.001^*^a
Sleep apnea	19 (8.2)	82 (3.4)	< 0.001^*^a	10 (4.7)	59 (2.3)	0.031^*^a
Smoker	61 (26.3)	822 (33.8)	0.020^*^a	32 (14.9)	571 (22.0)	0.014^*^a
**Medication history: No. (%)**						
HTN medication	185 (79.7)	1,578 (64.9)	< 0.001^*^a	186 (86.5)	1,845 (71.2)	< 0.001^*^a
Cholesterol reducer	134 (57.8)	1,093 (45.0)	< 0.001^*^a	128 (59.5)	1,073 (41.4)	< 0.001^*^a
Diabetic medication	99 (42.7)	636 (26.2)	< 0.001^*^a	80 (37.2)	680 (26.2)	< 0.001^*^a
Antidepressant	40 (17.2)	211 (8.7)	< 0.001^*^a	59 (27.4)	401 (15.5)	< 0.001^*^a
Antiplatelet prior	125 (53.9)	1,070 (44.0)	0.004^*^a	114 (53.0)	1,139 (43.9)	0.010^*^a
Anticoagulant prior	27 (11.6)	205 (8.4)	0.099^a^	24 (11.2)	207 (8.0)	0.103^a^
Initial NIHSS score	8.16 ± 8.679	7.58 ± 7.767	0.320^b^	8.56 ± 8.521	8.93 ± 8.564	0.570^b^
**Lab values: Mean** ±**SD**						
Total cholesterol, mg/dL	159.41 ± 49.566	166.55 ± 52.299	0.070^b^	169.77 ± 56.274	178.15 ± 50.415	0.036^*^b
Triglycerides, mg/dL	134.74 ± 88.651	143.31 ± 112.740	0.306^b^	157.59 ± 121.957	135.15 ± 96.998	0.004^*^b
HDL, mg/dL	37.46 ± 14.013	38.52 ± 12.432	0.264^b^	40.29 ± 12.465	45.40 ± 14.302	< 0.001^*^b
LDL, mg/dL	96.90 ± 39.768	102.29 ± 39.695	0.070^b^	101.86 ± 49.334	107.74 ± 41.985	0.079^b^
Lipids	6.75 ± 1.899	6.50 ± 1.813	0.072^b^	6.65 ± 1.891	6.54 ± 3.192	0.662^b^
Blood glucose, mg/dL	159.40 ± 100.146	145.53 ± 75.738	0.041^*^b	171.71 ± 108.342	145.82 ± 80.917	< 0.001^*^b
Serum creatinine, mg/dL	2.97 ± 2.393	1.26 ± 0.925	< 0.001^*^b	2.59 ± 2.178	1.06 ± 0.855	< 0.001^*^b
INR	1.19 ± 0.333	1.15 ± 0.525	0.407^b^	1.18 ± 0.617	1.12 ± 0.465	0.116^b^
**Vital signs: Mean** ±**SD**						
Heart rate, bpm	82.16 ± 19.091	80.26 ± 18.278	0.131^b^	84.62 ± 21.302	83.41 ± 18.329	0.417^b^
Blood pressure, systolic, mmHg	151.75 ± 30.784	152.13 ± 28.678	0.848^b^	150.61 ± 32.457	151.65 ± 29.508	0.625^b^
Blood pressure, diastolic, mmHg	81.19 ± 20.387	84.93 ± 18.138	0.003^*^b	77.73 ± 21.646	80.61 ± 19.397	0.038^*^b
**Ambulation status prior to event: No. (%)**						
Ambulate independently	194 (83.6)	2,259 (93.0)	< 0.001^*^a	173 (80.5)	2,261 (87.2)	0.005^*^a
Ambulate with assistance	10 (4.3)	64 (2.6)	0.138^a^	11 (5.1)	118 (4.6)	0.704^a^
Unable to ambulate	17 (7.3)	62 (2.6)	< 0.001^*^a	9 (4.2)	125 (4.8)	0.674^a^
Not documented	11 (4.7)	45 (1.9)	0.003^*^a	22 (10.2)	87 (3.4)	< 0.001^*^a
rtPA received	40 (17.2)	628 (25.8)	0.004^*^a	39 (18.1)	620 (23.9)	0.055^a^
First care: emergency department	36 (15.5)	514 (21.2)	0.038^*^a	38 (17.7)	536 (20.7)	0.269^a^
Diastolic BP ≥ 80 mmHg	121 (52.2)	1,455 (59.9)	0.021^*^a	87 (40.5)	1,225 (47.3)	0.052^a^

Continuous variables are presented as mean ± standard deviation (SD), and categorical variables as *n* (%). Comparisons were performed within each sex stratum using Pearson's chi-square test for categorical variables and Student's *t* test for continuous variables.

a Pearson's chi-square test.

b Student's t-test.

* *P*-value < 0.05.

### 3.2 Factors independently associated with CKD in the overall AIS population

In the multivariable model with documented CKD as the outcome, several clinical characteristics were independently associated with CKD (Table 3; Figure 1). Higher serum creatinine was strongly associated with CKD (OR, 2.786; 95% CI, 2.389–3.249; *P* < 0.001). African American race, compared with White race, was also associated with CKD (OR, 2.106; 95% CI, 1.485–2.987; *P* < 0.001). Other positive associations were observed for prosthetic heart valve (OR, 3.728; 95% CI, 1.645–8.448; *P* = 0.002), obesity (OR, 2.509; 95% CI, 1.816–3.466; *P* < 0.001), heart failure (OR, 2.214; 95% CI, 1.542–3.178; *P* < 0.001), hypertension (OR, 2.201; 95% CI, 1.263–3.837; *P* = 0.005), depression (OR, 1.971; 95% CI, 1.375–2.825; *P* < 0.001), peripheral vascular disease (OR, 1.828; 95% CI, 1.182–2.827; *P* = 0.007), and increasing age (OR, 1.018; 95% CI, 1.005–1.030; *P* = 0.006). In contrast, requiring assistance with ambulation was inversely associated with documented CKD (OR, 0.597; 95% CI, 0.409–0.873; *P* = 0.008).

**Table 3:** Multivariable and bootstrap-validated clinical characteristics associated with documented chronic kidney disease among patients with acute ischemic stroke.

Variable	Original OR (95% CI)	*P*	Bootstrap OR	Bootstrap 95% CI	Selection frequency
Age	1.018 (1.005–1.030)	0.006	1.018	1.004–1.031	82%
Total cholesterol	0.997 (0.994–1.000)	0.070	0.997	0.993–1.001	48%
Blood glucose	1.001 (1.000–1.003)	0.088	1.001	0.999–1.003	43%
Serum creatinine	2.786 (2.389–3.249)	< 0.001	2.80	2.37–3.32	100%
African American vs. Caucasian	2.106 (1.485–2.987)	< 0.001	2.10	1.45–3.05	97%
Other vs. Caucasian	0.263 (0.048–1.437)	0.123	0.29	0.04–1.62	38%
Atrial fibrillation	1.380 (0.963–1.976)	0.079	1.38	0.94–2.04	46%
Carotid artery stenosis	1.594 (0.971–2.614)	0.065	1.59	0.92–2.75	52%
Depression	1.971 (1.375–2.825)	< 0.001	1.96	1.33–2.91	94%
Dyslipidemia	1.369 (0.994–1.886)	0.055	1.37	0.97–1.94	55%
Heart failure	2.214 (1.542–3.178)	< 0.001	2.20	1.49–3.27	96%
Hypertension	2.201 (1.263–3.837)	0.005	2.19	1.20–4.03	83%
Obesity	2.509 (1.816–3.466)	< 0.001	2.50	1.77–3.54	99%
Previous stroke	1.337 (0.982–1.820)	0.065	1.34	0.96–1.87	52%
Prosthetic heart valve	3.728 (1.645–8.448)	0.002	3.67	1.42–9.32	78%
Peripheral vascular disease	1.828 (1.182–2.827)	0.007	1.82	1.13–2.95	82%
Ambulate with assistance	0.597 (0.409–0.873)	0.008	0.60	0.40–0.91	79%
Unable to ambulate	0.733 (0.518–1.039)	0.081	0.74	0.50–1.08	45%
Diastolic BP ≥80 mmHg	0.741 (0.548–1.002)	0.052	0.74	0.53–1.03	57%

An adjusted odds ratio (OR) >1 indicates an association with documented CKD, whereas an OR < 1 indicates an association with no documented CKD. Bootstrap ORs, 95% confidence intervals (CIs), and selection frequencies are provided as measures of internal stability. Selection frequency represents the percentage of bootstrap samples in which a variable was retained in the final model. Hosmer–Lemeshow test, *P* = 0.041; Cox and Snell R^2^ = 0.146. *P* < 0.05.

**Figure 1:**
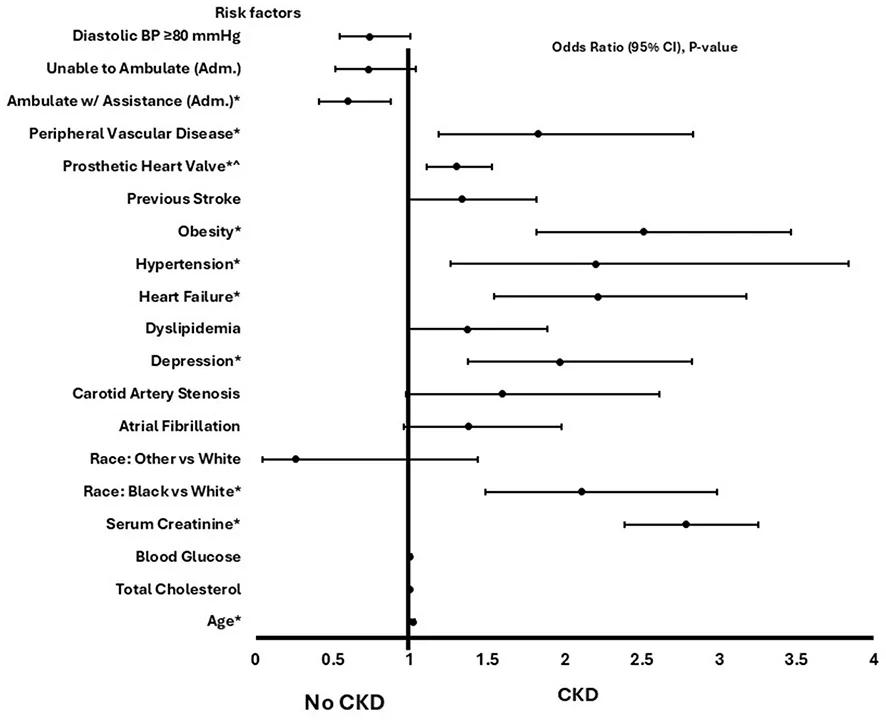
Forest Plot representation of Table 3. Confidence interval band below 1 denotes factors that are associated with not having CKD while Confidence interval band above 1 denotes factors that are associated with having CKD. Confidence interval bands that cross 1 cannot be associated with either having or not having CKD. *Indicates statistical significance (*P* < 0.05) with a 95% confidence interval. ∧Indicates that data were modified by taking the 5th square root.

Bootstrap validation was based on 1,000 resamples, with selection frequency defined as the percentage of resamples in which a variable was retained in the final model. Serum creatinine was the most stable factor, with a bootstrap OR of 2.80 (95% CI, 2.37–3.32) and a 100% selection frequency. High selection frequencies were also observed for obesity (bootstrap OR, 2.50; 95% CI, 1.77–3.54; 99%), African American race (OR, 2.10; 95% CI, 1.45–3.05; 97%), heart failure (OR, 2.20; 95% CI, 1.49–3.27; 96%), and depression (OR, 1.96; 95% CI, 1.33–2.91; 94%). Hypertension (OR, 2.19; 95% CI, 1.20–4.03; 83%), increasing age (OR, 1.018; 95% CI, 1.004–1.031; 82%), peripheral vascular disease (OR, 1.82; 95% CI, 1.13–2.95; 82%), and ambulation with assistance (OR, 0.60; 95% CI, 0.40–0.91; 79%) also showed relative stability. Prosthetic heart valve retained a positive association (bootstrap OR, 3.67; 95% CI, 1.42–9.32; 78%), although its wide confidence interval indicated greater uncertainty.

Total cholesterol, blood glucose, atrial fibrillation, carotid artery stenosis, dyslipidemia, previous stroke, inability to ambulate, other race vs. White race, and DBP ≥80 mmHg had lower selection frequencies (38%−57%), and their bootstrap confidence intervals crossed the null. These findings indicated greater sensitivity to sampling variability. The model had an AUC of 0.663 (95% CI, 0.632–0.694) and a Cox and Snell R^2^ of 0.146. The Hosmer–Lemeshow test was statistically significant (*P* = 0.041), indicating some departure from calibration. Although overall classification accuracy was 93.0%, this measure was interpreted cautiously because only 8.2% of patients had CKD and accuracy was therefore strongly influenced by the much larger non-CKD group. Model performance (Figure 2) was consequently assessed primarily using discrimination, calibration, and stability of the estimated associations.

**Figure 2:**
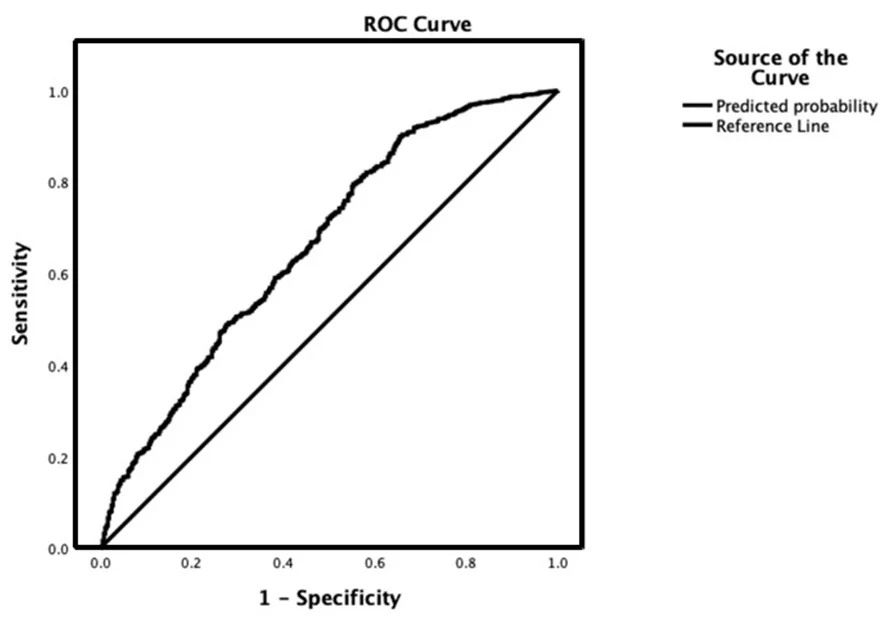
ROC curve associated with prediction of chronic kidney disease (CKD) in the whole acute ischemic stroke population. Higher area under the curve (AUC) values in ROC analysis indicate better discrimination of the score for the measured outcome. Classification table (overall correctly classified percentage = 93.0%) and area under the ROC curve (AUC = 0.663, 0.632–0.694) were applied.

### 3.3 Clinical characteristics independently associated with sex among AIS patients without CKD

Among patients without CKD, the multivariable model identified several characteristics associated with male sex (Table 4; Figure 3). These included serum creatinine (OR, 2.856; 95% CI, 2.264–3.603; *P* < 0.001), sleep apnea (OR, 2.377; 95% CI, 1.454–3.887; *P* < 0.001), coronary artery disease (OR, 1.777; 95% CI, 1.476–2.140; *P* < 0.001), independent ambulation before the event (OR, 1.876; 95% CI, 1.350–2.608; *P* < 0.001), DBP ≥80 mmHg (OR, 1.427; 95% CI, 1.117–1.824; *P* = 0.004), obesity (OR, 1.397; 95% CI, 1.160–1.682; *P* < 0.001), smoking (OR, 1.313; 95% CI, 1.081–1.594; *P* = 0.006), and elevated INR (OR, 1.339; 95% CI, 1.051–1.705; *P* = 0.018). Higher continuous DBP was also associated with male sex (OR, 1.017; 95% CI, 1.010–1.024; *P* < 0.001). Age group was significant overall (*P* < 0.001), with greater odds of male sex among patients aged 50–59, 60–69, and 70–79 years than among those younger than 50 years. In contrast, age modeled continuously was inversely associated with male sex (OR, 0.974; 95% CI, 0.955–0.994; *P* = 0.012).

**Table 4:** Multivariable and bootstrap-validated clinical characteristics associated with male sex among patients with acute ischemic stroke without documented chronic kidney disease.

Variable	Original OR (95% CI)	*P* value	Bootstrap OR	Bootstrap 95% CI	Selection frequency
Age	0.974 (0.955–0.994)	0.012	0.974	0.954–0.995	76%
BMI	0.957 (0.944–0.971)	< 0.001	0.957	0.943–0.971	99%
Total cholesterol	0.994 (0.992–0.996)	< 0.001	0.994	0.992–0.996	100%
HDL	0.969 (0.962–0.975)	< 0.001	0.969	0.962–0.976	100%
Serum creatinine	2.856 (2.264–3.603)	< 0.001	2.84	2.23–3.65	100%
INR	1.339 (1.051–1.705)	0.018	1.33	1.03–1.72	72%
Heart rate	0.982 (0.977–0.987)	< 0.001	0.982	0.977–0.987	100%
Diastolic BP, continuous	1.017 (1.010–1.024)	< 0.001	1.017	1.010–1.024	98%
Age group (overall)	—	< 0.001	—	—	96%
50–59 vs. < 50	2.556 (1.728–3.779)	< 0.001	2.53	1.68–3.84	97%
60–69 vs. < 50	2.847 (1.668–4.859)	< 0.001	2.81	1.61–4.94	94%
70–79 vs. < 50	2.689 (1.315–5.501)	0.007	2.65	1.25–5.64	80%
≥80 vs. < 50	1.866 (0.740–4.708)	0.186	1.82	0.68–4.86	31%
Race group (overall)	—	0.012	—	—	74%
African American vs. Caucasian	0.725 (0.580–0.906)	0.005	0.727	0.574–0.918	83%
Other vs. Caucasian	0.758 (0.488–1.176)	0.216	0.77	0.47–1.25	28%
Coronary artery disease	1.777 (1.476–2.140)	< 0.001	1.77	1.46–2.15	100%
Carotid artery stenosis	1.404 (0.974–2.025)	0.069	1.40	0.95–2.07	51%
Depression	0.601 (0.402–0.897)	0.013	0.61	0.40–0.93	76%
Heart failure	0.658 (0.491–0.882)	0.005	0.66	0.48–0.90	83%
Obesity	1.397 (1.160–1.682)	< 0.001	1.39	1.14–1.70	96%
Peripheral vascular disease	0.751 (0.535–1.054)	0.097	0.76	0.52–1.10	43%
Sleep apnea	2.377 (1.454–3.887)	< 0.001	2.34	1.39–3.96	91%
Smoking	1.313 (1.081–1.594)	0.006	1.31	1.06–1.62	84%
HTN medication	0.706 (0.586–0.851)	< 0.001	0.71	0.58–0.87	95%
Antidepressant	0.590 (0.395–0.883)	0.010	0.60	0.39–0.92	79%
Independent ambulation, prior	1.876 (1.350–2.608)	< 0.001	1.86	1.32–2.63	94%
Unable to ambulate, admission	0.769 (0.641–0.923)	0.005	0.77	0.63–0.94	85%
rtPA received	1.172 (0.985–1.394)	0.073	1.17	0.97–1.41	49%
Diastolic BP ≥80 mmHg	1.427 (1.117–1.824)	0.004	1.42	1.09–1.85	86%

An adjusted OR >1 indicates an association with male sex, whereas an OR < 1 indicates an association with female sex. Bootstrap ORs, 95% CIs, and selection frequencies are provided as measures of internal stability. Selection frequency represents the percentage of bootstrap samples in which a variable was retained in the final model. Hosmer–Lemeshow test, *P* = 0.178; Cox and Snell R^2^ = 0.216. *P* < 0.05.

**Figure 3:**
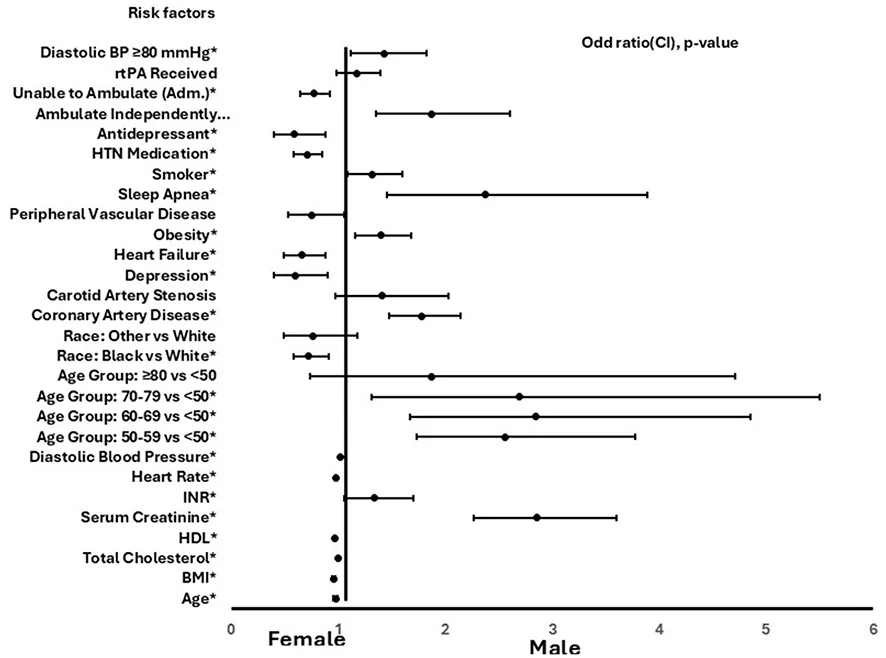
Forest Plot representation of Table 4. Confidence interval band below 1 denotes factors that are associated with being female while Confidence interval band above 1 denotes factors that are associated with being male among acute ischemic stroke patients without CKD. Confidence interval bands that cross 1 cannot be associated with either gender. *Indicates statistical significance (*P* < 0.05) with a 95% confidence interval.

Characteristics associated with lower odds of male sex included higher HDL cholesterol (OR, 0.969; 95% CI, 0.962–0.975; *P* < 0.001), BMI (OR, 0.957; 95% CI, 0.944–0.971; *P* < 0.001), total cholesterol (OR, 0.994; 95% CI, 0.992–0.996; *P* < 0.001), and heart rate (OR, 0.982; 95% CI, 0.977–0.987; *P* < 0.001). Lower odds were also observed for heart failure (OR, 0.658; 95% CI, 0.491–0.882; *P* = 0.005), depression (OR, 0.601; 95% CI, 0.402–0.897; *P* = 0.013), antidepressant use (OR, 0.590; 95% CI, 0.395–0.883; *P* = 0.010), antihypertensive medication use (OR, 0.706; 95% CI, 0.586–0.851; *P* < 0.001), African American race relative to White race (OR, 0.725; 95% CI, 0.580–0.906; *P* = 0.005), and inability to ambulate at admission (OR, 0.769; 95% CI, 0.641–0.923; *P* = 0.005).

Bootstrap validation supported the stability of most principal associations. Serum creatinine, total cholesterol, HDL cholesterol, heart rate, and coronary artery disease were selected in 100% of bootstrap samples. High selection frequencies were also observed for BMI (99%), continuous DBP (98%), obesity (96%), antihypertensive medication use (95%), independent pre-event ambulation (94%), and sleep apnea (91%). Bootstrap estimates closely approximated the original estimates, with confidence intervals excluding the null. Smoking (bootstrap OR, 1.31; 95% CI, 1.06–1.62; selection frequency, 84%), heart failure (OR, 0.66; 95% CI, 0.48–0.90; 83%), inability to ambulate at admission (OR, 0.77; 95% CI, 0.63–0.94; 85%), antidepressant use (OR, 0.60; 95% CI, 0.39–0.92; 79%), depression (OR, 0.61; 95% CI, 0.40–0.93; 76%), and INR (OR, 1.33; 95% CI, 1.03–1.72; 72%) also showed bootstrap support.

The age-group effect was retained in 96% of bootstrap samples. Compared with patients younger than 50 years, the associations remained stable for ages 50–59 years (bootstrap OR, 2.53; 95% CI, 1.68–3.84; 97%), 60–69 years (OR, 2.81; 95% CI, 1.61–4.94; 94%), and 70–79 years (OR, 2.65; 95% CI, 1.25–5.64; 80%). The association for age ≥80 years was less stable (OR, 1.82; 95% CI, 0.68–4.86; 31%). The African American vs. White association was also relatively stable (bootstrap OR, 0.727; 95% CI, 0.574–0.918; 83%).

Carotid artery stenosis (51%), rtPA receipt (49%), peripheral vascular disease (43%), and other race vs. White race (28%) had lower selection frequencies, with bootstrap confidence intervals that included 1.0. Overall, bootstrap validation supported the reproducibility of most principal sex-associated characteristics while identifying several weaker associations as sensitive to sampling variability. Model performance (Figure 4) was assessed primarily using discrimination, calibration, and stability of the estimated associations.

**Figure 4:**
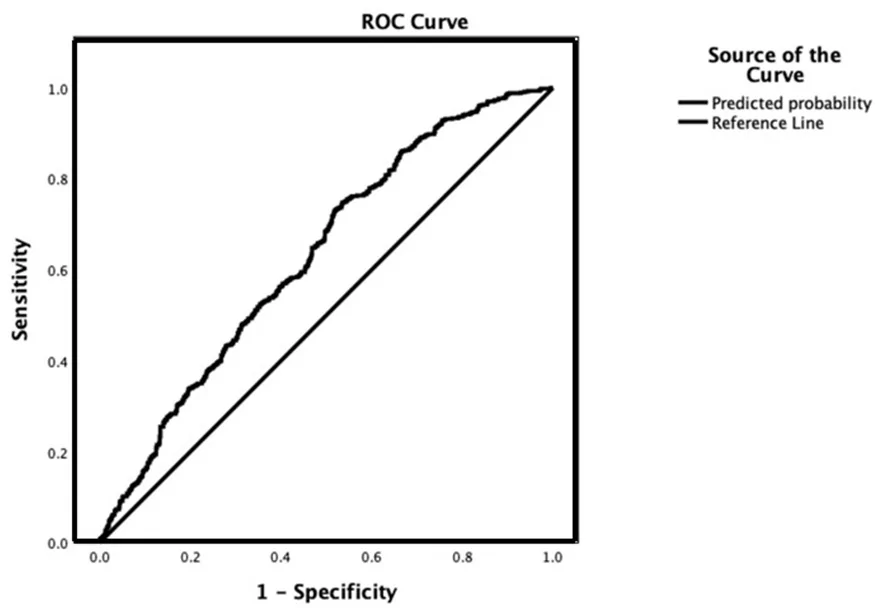
ROC curve associated with acute ischemic stroke patients without CKD. Higher area under the curve (AUC) values in ROC analysis indicate better discrimination of the score for the measured outcome. Classification table (overall correctly classified percentage = 70.3%) and area under the ROC curve (AUC = 0.630, 0.599–0.661) were applied to check model fitness.

### 3.4 Clinical characteristics associated with sex among AIS patients with CKD

Among patients with CKD, the multivariable model identified several characteristics associated with male sex (Table 5; Figure 5). These included prosthetic heart valve (OR, 6.450; 95% CI, 1.058–39.329; *P* = 0.043), sleep apnea (OR, 4.616; 95% CI, 1.361–15.652; *P* = 0.014), independent ambulation at admission (OR, 3.472; 95% CI, 1.437–8.385; *P* = 0.005), smoking (OR, 3.192; 95% CI, 1.330–7.664; *P* = 0.009), DBP ≥80 mmHg (OR, 2.581; 95% CI, 1.295–5.062; *P* = 0.006), dyslipidemia (OR, 2.262; 95% CI, 1.098–4.657; *P* = 0.027), and higher LDL cholesterol (OR, 1.040; 95% CI, 1.015–1.066; *P* = 0.002). Characteristics associated with lower odds of male sex included hypertension (OR, 0.262; 95% CI, 0.072–0.952; *P* = 0.042), depression (OR, 0.408; 95% CI, 0.186–0.892; *P* = 0.025), higher total cholesterol (OR, 0.966; 95% CI, 0.946–0.985; *P* < 0.001), higher BMI (OR, 0.930; 95% CI, 0.888–0.975; *P* = 0.003), higher blood glucose (OR, 0.996; 95% CI, 0.992–0.999; *P* = 0.026), and older age (OR, 0.956; 95% CI, 0.928–0.986; *P* = 0.004). Model performance (Figure 6) was assessed primarily using discrimination, calibration, and stability of the estimated associations.

**Table 5:** Multivariable and bootstrap-validated clinical characteristics associated with male sex among patients with acute ischemic stroke with documented chronic kidney disease.

Variable	Original OR (95% CI)	*P* value	Bootstrap OR	Bootstrap 95% CI	Selection frequency	Firth OR
Age	0.956 (0.928–0.986)	0.004	0.957	0.926–0.988	83%	~0.958
BMI	0.930 (0.888–0.975)	0.003	0.932	0.885–0.979	85%	~0.934
Total cholesterol	0.966 (0.946–0.985)	< 0.001	0.966	0.944–0.987	91%	~0.968
LDL	1.040 (1.015–1.066)	0.002	1.039	1.012–1.068	87%	~1.037
Blood glucose	0.996 (0.992–0.999)	0.026	0.996	0.991–1.000	68%	~0.997
Heart rate	0.983 (0.965–1.002)	0.084	0.984	0.963–1.005	45%	~0.985
Depression	0.408 (0.186–0.892)	0.025	0.421	0.174–0.960	69%	~0.44
Dyslipidemia	2.262 (1.098–4.657)	0.027	2.20	1.01–4.89	66%	~2.10
Hypertension	0.262 (0.072–0.952)	0.042	0.278	0.065–1.09	59%	~0.31
Prosthetic heart valve	6.450 (1.058–39.329)	0.043	5.92	0.72–45.8	52%	~4.5–5.5
Sleep apnea	4.616 (1.361–15.652)	0.014	4.42	1.18–17.3	72%	~4.0–4.3
Smoking	3.192 (1.330–7.664)	0.009	3.12	1.24–8.02	78%	~2.9–3.1
Diabetic medication	1.930 (0.788–3.522)	0.065	1.88	0.73–3.89	48%	~1.7–1.8
Independent ambulation, admission	3.472 (1.437–8.385)	0.005	3.38	1.31–8.91	81%	~3.1–3.3
Independent ambulation, discharge	0.455 (0.218–1.025)	0.043	0.47	0.20–1.08	60%	~0.50
First care: emergency department	0.446 (0.178–1.047)	0.069	0.46	0.17–1.17	46%	~0.50
Diastolic BP ≥80 mmHg	2.581 (1.295–5.062)	0.006	2.52	1.21–5.35	80%	~2.4–2.5

An adjusted OR >1 indicates an association with male sex, whereas an OR < 1 indicates an association with female sex. Bootstrap ORs, 95% CIs, and selection frequencies are provided as measures of internal stability. The area under the receiver operating characteristic curve (AUC) represents apparent within-sample discrimination. Hosmer–Lemeshow test, *P* = 0.139; Cox and Snell R^2^ = 0.312; overall correct classification, 76.0%. Three pre-event ambulation variables—independent ambulation, ambulation with assistance, and inability to ambulate—exhibited complete separation and were excluded from the final model. *P* < 0.05.

**Figure 5:**
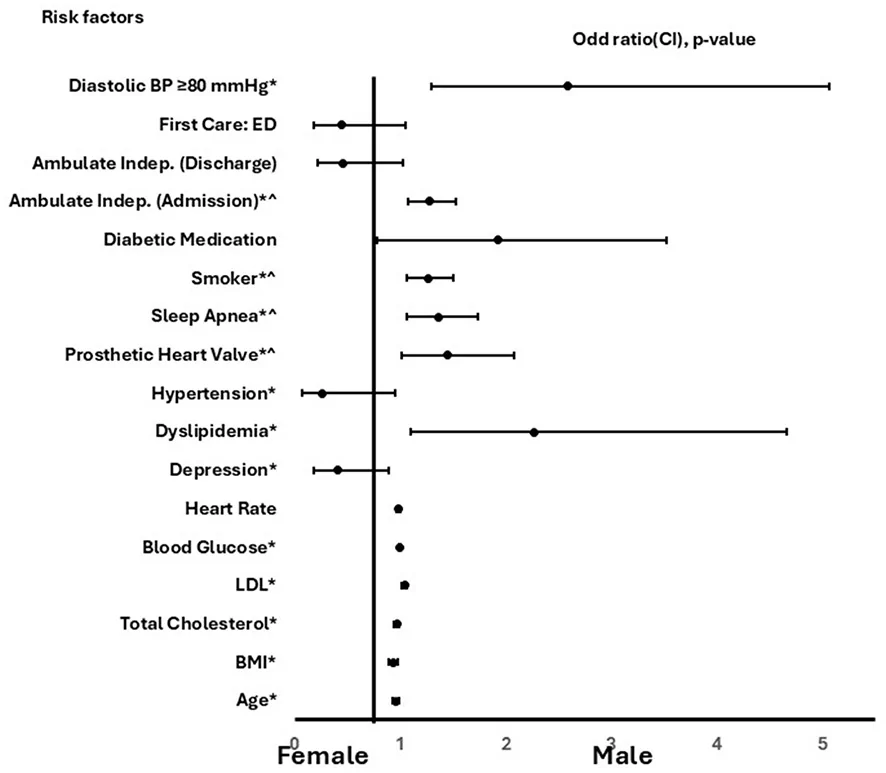
Forest Plot representation of Table 5. Confidence interval band below 1 denotes factors that are associated with being female while Confidence interval band above 1 denotes factors that are associated with being male among acute ischemic stroke patients with CKD. Confidence interval bands that cross 1 cannot be associated with either gender. *Indicates statistical significance (*P* < 0.05) with a 95% confidence interval. ∧Indicates that data were modified by taking the 5th square root.

**Figure 6:**
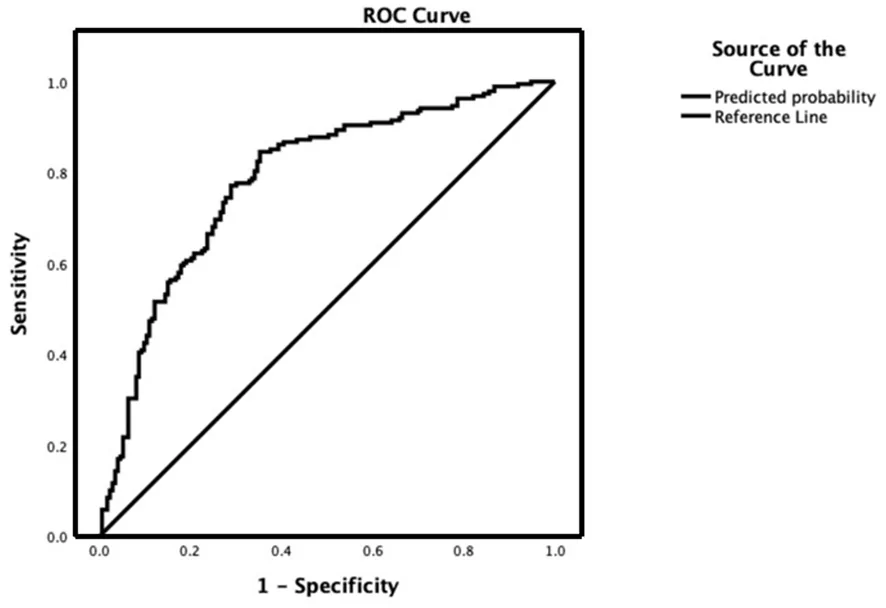
ROC curve associated with acute ischemic stroke patients with CKD. Higher area under the curve (AUC) values in ROC analysis indicate better discrimination of the score for the measured outcome. Classification table (overall correctly classified percentage = 76%) and area under the ROC curve (AUC = 0.785, 0.737–0.832) were applied to check model fitness.

Bootstrap validation provided support for several associations. Total cholesterol had the highest selection frequency (91%; bootstrap OR, 0.966; 95% CI, 0.944–0.987), followed by LDL cholesterol (87%; OR, 1.039; 95% CI, 1.012–1.068), BMI (85%; OR, 0.932; 95% CI, 0.885–0.979), and age (83%; OR, 0.957; 95% CI, 0.926–0.988). Independent ambulation at admission (81%; OR, 3.38; 95% CI, 1.31–8.91), DBP ≥80 mmHg (80%; OR, 2.52; 95% CI, 1.21–5.35), smoking (78%; OR, 3.12; 95% CI, 1.24–8.02), and sleep apnea (72%; OR, 4.42; 95% CI, 1.18–17.3) also showed relative stability. Depression (69%; OR, 0.421; 95% CI, 0.174–0.960) and dyslipidemia (66%; OR, 2.20; 95% CI, 1.01–4.89) had moderate bootstrap support.

Blood glucose was selected in 68% of bootstrap samples, but its confidence interval reached the null (OR, 0.996; 95% CI, 0.991–1.000). Hypertension had a 59% selection frequency, with a bootstrap confidence interval crossing 1.0 (OR, 0.278; 95% CI, 0.065–1.09). Although prosthetic heart valve had the largest original point estimate, it was selected in only 52% of bootstrap samples and remained imprecise (bootstrap OR, 5.92; 95% CI, 0.72–45.8), suggesting potential sparse-data instability. Heart rate (45%), diabetic medication use (48%), first care in the Emergency Department (46%), and independent ambulation at discharge (60%) also showed lower stability, with bootstrap confidence intervals including the null. Overall, the strongest bootstrap support was observed for age, BMI, total cholesterol, LDL cholesterol, independent ambulation at admission, elevated DBP, smoking, and sleep apnea. Less stable estimates warrant cautious interpretation.

Sleep apnea, smoking, and DBP ≥80 mmHg were associated with male sex in both CKD strata, with numerically larger point estimates among patients with CKD. In pooled models incorporating CKD × clinical-characteristic interaction terms, the interaction ORs were 1.94 for sleep apnea (P interaction = 0.323), 2.43 for smoking (P interaction = 0.052), and 1.81 for DBP ≥80 mmHg (P interaction = 0.109). None reached the prespecified threshold for statistical significance. Thus, the between-stratum differences were descriptive rather than evidence of statistically significant effect modification. The CKD × smoking interaction approached statistical significance and should be considered hypothesis-generating.

### 3.5 Model performance and sensitivity analyses

The whole-cohort CKD model demonstrated modest apparent discrimination (AUC, 0.663; 95% CI, 0.632–0.694), with a Cox and Snell R^2^ of 0.146. The Hosmer–Lemeshow test was statistically significant (*P* = 0.041), indicating some departure from calibration. Overall classification accuracy was 93.0%; however, this measure was not emphasized because CKD represented only 8.2% of the cohort and accuracy was therefore strongly influenced by class imbalance. The apparent AUCs of the sex-associated models were 0.630 (95% CI, 0.599–0.661) in the non-CKD subgroup and 0.785 (95% CI, 0.737–0.832) in the CKD subgroup. Because these models were developed independently in different strata with different predictor sets, the AUCs were interpreted as within-sample measures of discrimination rather than evidence of superior performance or greater biological differentiation in the CKD subgroup. Bootstrap internal validation and Firth bias-reduced logistic regression were used to assess the robustness of the CKD-stratified model. Bootstrap validation showed relatively high stability for total cholesterol (selection frequency, 91%), LDL cholesterol (87%), BMI (85%), age (83%), independent ambulation at admission (81%), DBP ≥80 mmHg (80%), smoking (78%), and sleep apnea (72%). Bootstrap estimates for these variables closely approximated the conventional estimates. Firth regression generally preserved their direction and approximate magnitude, with modest shrinkage toward the null, supporting their relative robustness to small-sample bias.

In contrast, prosthetic heart valve demonstrated substantial instability. Although conventional logistic regression yielded an OR of 6.450 (95% CI, 1.058–39.329), the bootstrap estimate was 5.92 (95% CI, 0.72–45.8), with a selection frequency of only 52%; the Firth estimate was also attenuated toward the null. Hypertension and blood glucose showed weaker bootstrap stability and attenuation under penalization. Associations supported by both bootstrap validation and Firth regression therefore appeared more robust, whereas estimates affected by sparse observations require cautious interpretation.

## 4 Discussion

The principal contribution of this study is the integrated evaluation of sex-associated clinical profiles according to CKD status among patients with established AIS. Previous studies have separately examined sex differences in AIS and sex-related differences in CKD recognition, progression, and cerebrovascular risk. However, these bodies of literature [(11, 22)] have generally evaluated sex and renal status independently rather than characterizing the multivariable clinical profiles associated with sex within CKD and non-CKD AIS strata. By comparing these profiles within the same AIS cohort, we identified differences in the demographic, vascular, metabolic, functional, and clinical characteristics associated with male vs. female sex according to renal status. A further contribution is the use of formal interaction testing to distinguish descriptive differences between CKD-stratified models from true statistical effect modification. Although sleep apnea, smoking, and DBP ≥80 mmHg had numerically stronger associations with male sex among patients with CKD, pooled models incorporating CKD × clinical-characteristic interaction terms did not demonstrate statistically significant heterogeneity for any of these factors. Thus, this study does not establish that CKD universally modifies sex-associated relationships. Rather, it demonstrates how sex-associated clinical profiles vary across renal-status strata while formally testing whether selected associations differ according to CKD status.

Three principal findings emerged. First, CKD was associated with a distinct comorbidity profile among patients with AIS, characterized by older age, African American race, and a greater burden of cardiovascular and metabolic risk factors. Second, among patients without CKD, sex-associated clinical characteristics were broadly consistent with the established stroke literature [(11, 22), 32]. Third, the composition and magnitude of sex-associated characteristics differed descriptively across CKD strata. Although several associations had larger effect estimates among patients with CKD, formal interaction analyses did not establish statistically significant CKD-dependent heterogeneity for sleep apnea, smoking, or elevated diastolic blood pressure. These findings therefore support differences in CKD-stratified clinical profiles but not definitive effect modification by CKD.

### 4.1 The CKD phenotype in acute ischemic stroke

The independent association between CKD and African Americans in our cohort is consistent with well-documented disparities in CKD prevalence and progression (19, 20) and underscores the importance of considering renal status in demographically heterogeneous stroke populations. The concentration of cardiovascular and metabolic comorbidities among patients with CKD—including heart failure, hypertension, diabetes, dyslipidemia, obesity, and peripheral vascular disease—suggests a shared vascular phenotype linking renal dysfunction and cerebrovascular disease. This pattern may reflect common exposure of the kidney and brain to hemodynamic and atherosclerotic injury, thereby contributing to small-vessel disease in both organs (6, 8). The lower prevalence of smoking among patients with CKD, although initially unexpected, may reflect their older age distribution and greater clinical surveillance. Similar patterns have been reported in other comorbid stroke cohorts (23).

### 4.2 Sex-associated clinical characteristics in the absence of CKD

Among patients without CKD, the sex-associated clinical characteristics were generally consistent with prior studies of sex differences in stroke (12). Female patients were more likely to have higher HDL and total cholesterol levels, higher BMI, depression, antidepressant use, and antihypertensive treatment. Male patients were more likely to have coronary artery disease, smoking, sleep apnea, and elevated diastolic blood pressure. Thus, in the absence of CKD, the distribution of vascular and clinical factors followed patterns previously reported in larger stroke populations (12, 13).

### 4.3 Sex-associated clinical characteristics in the presence of CKD

Formal interaction analyses clarified the differences observed between CKD strata. The interaction ORs were 1.94 for sleep apnea, 2.43 for smoking, and 1.81 for DBP ≥80 mmHg, indicating that the corresponding associations with male sex were numerically stronger among patients with CKD. However, none of these interactions reached statistical significance (P interaction = 0.323, 0.052, and 0.109, respectively). These findings therefore do not provide sufficient evidence that CKD significantly modifies the associations of sleep apnea, smoking, or elevated DBP with sex. The CKD × smoking interaction was closest to statistical significance and warrants evaluation in larger cohorts with greater power to detect interaction effects.

Serum creatinine, coronary artery disease, and HDL cholesterol were independent sex-associated factors among patients without CKD but were not retained in the CKD model. The serum creatinine findings warrant particular consideration. Higher serum creatinine was strongly associated with documented CKD in the overall AIS cohort, as expected given its relationship with renal filtration and clinical recognition of renal dysfunction. Although serum creatinine was not used by the investigators to define CKD, renal-function measurements may have contributed to the clinical diagnoses subsequently recorded in the registry. Accordingly, this association should not be interpreted as identifying serum creatinine as a novel determinant of CKD.

Serum creatinine was also strongly associated with male sex among patients without documented CKD. This finding likely reflects established physiological sex differences in creatinine generation. Serum creatinine is influenced by skeletal muscle mass as well as glomerular filtration, and men generally have greater muscle mass and higher serum creatinine concentrations than women at comparable levels of renal filtration (17, 18). Thus, the observed associations may reflect both renal-function ascertainment and sex-related physiology rather than a novel CKD- or AIS-specific relationship.

These considerations also highlight the limitations of using serum creatinine alone to characterize renal function. Because the registry lacked longitudinal eGFR, albuminuria, CKD stage, and dialysis status, we could not determine whether elevated creatinine among patients without documented CKD represented unrecognized chronic renal impairment, acute kidney injury, physiological variation in creatinine generation, or a combination of these factors. Future studies incorporating standardized, longitudinal measures of kidney function are needed to clarify these relationships.

Conversely, dyslipidemia, LDL cholesterol, hypertension, and blood glucose emerged as independent sex-associated characteristics specifically among patients with CKD. These findings suggest that the characteristics distinguishing male from female patients may vary descriptively according to renal status, without establishing CKD as a statistical effect modifier of individual risk-factor associations.

### 4.4 Comparison of sex-associated profiles across CKD strata

Documented CKD was present in 8.2% of the AIS cohort, a prevalence lower than that reported in several stroke populations in which renal dysfunction is systematically identified using eGFR-based criteria. This difference may partly reflect reliance on registry-documented clinical CKD rather than systematic laboratory-based ascertainment. Patients with mild, previously unrecognized, or incompletely documented CKD may therefore have been classified in the non-CKD group.

Patients with documented CKD had substantially higher serum creatinine levels than those without CKD (2.79 ± 2.30 vs. 1.16 ± 0.90 mg/dL), supporting greater renal dysfunction in the documented CKD group. However, this difference does not validate classification across the full spectrum of CKD severity. Although the whole-cohort CKD model correctly classified 93.0% of observations, CKD accounted for only 8.2% of the cohort. Consequently, overall accuracy was driven largely by classification of the majority non-CKD group and does not establish strong discrimination of CKD cases.

The model demonstrated modest discrimination (AUC, 0.663), and the significant Hosmer–Lemeshow test (*P* = 0.041) suggested some departure from calibration. Overall classification accuracy was therefore not considered a primary measure of model performance. The CKD and non-CKD models also differed in their apparent discriminatory performance: the AUC was 0.785 among patients with CKD and 0.630 among those without CKD, with corresponding Cox and Snell R^2^ values of 0.312 and 0.216. These findings indicate that the measured clinical variables collectively differentiated male from female patients within the CKD subgroup. However, differences in model discrimination should not be interpreted as evidence of statistical interaction or causal modification by CKD.

Taken together, the results distinguish differences in multivariable clinical profiles from individual effect modification. Formal interaction testing did not establish significant CKD-dependent modification of the associations between sex and sleep apnea, smoking, or elevated diastolic blood pressure. Nevertheless, the stratified models differed in the magnitude of several effect estimates, the variables retained as independent correlates of sex, and their overall discriminatory performance. AIS patients with CKD may therefore exhibit a clinically distinct constellation of sex-associated vascular and metabolic characteristics, although CKD was not shown to modify individual associations statistically.

The near-significant interaction observed for smoking (P interaction = 0.052) warrants further investigation. Smoking was associated with male sex in both groups, but the effect estimate was substantially larger among patients with CKD than among those without CKD (OR, 3.192 vs. 1.313). The relatively small CKD subgroup and wider confidence intervals may have limited the precision required to detect interaction effects. This finding should therefore be considered exploratory and evaluated in larger cohorts with sufficient power for prespecified interaction analyses. The apparent difference in model discrimination between the CKD and non-CKD groups should also be interpreted cautiously. Because the models were fitted independently in different strata and included different predictor sets, their AUCs are not directly comparable measures of CKD-dependent biological heterogeneity. The higher AUC in the CKD subgroup may reflect differences in case mix, predictor composition, sample characteristics, or model optimism, particularly given the smaller CKD sample. Accordingly, these AUCs should be interpreted only as measures of within-sample discrimination; they do not demonstrate that CKD produces a stronger or more distinct sex-associated biological structure.

Bootstrap analyses provided additional information about the stability of the multivariable findings. Several principal associations demonstrated high selection frequencies, and their bootstrap estimates closely approximated the original estimates, supporting relative reproducibility within the present dataset. Other variables had lower selection frequencies or bootstrap confidence intervals that crossed the null, indicating greater sensitivity to sampling variability. This distinction was particularly evident in the CKD subgroup, in which the prosthetic-heart-valve association had a large point estimate but only a 52% bootstrap selection frequency and a very wide bootstrap confidence interval.

## 5 Conclusion

This study provides an integrated characterization of sex-associated clinical profiles among patients with AIS according to CKD status. Although the composition and magnitude of several associations differed descriptively between CKD strata, formal interaction testing did not demonstrate significant CKD-dependent modification of the principal shared associations examined. These findings extend prior literature by examining the intersection of sex and renal status in established AIS while distinguishing clinical-profile heterogeneity from formal effect modification. The results are exploratory and require confirmation in larger, independent cohorts.

## 6 Limitations

Several limitations should be considered. First, this was a retrospective, single-registry analysis; therefore, the reported associations cannot establish causality. Second, CKD status was ascertained from documented and coded diagnoses rather than longitudinal eGFR trajectories. Because CKD documentation may be less complete in women than in men (17, 18), some patients with unrecognized renal dysfunction may have been classified in the non-CKD group, and this misclassification may not have been sex-neutral. The observed CKD prevalence of 8.2% may therefore underestimate the underlying prevalence of renal dysfunction, particularly among patients with mild or previously unrecognized CKD. Future studies incorporating longitudinal eGFR, albuminuria, CKD stage, and dialysis status are needed to validate these CKD-stratified findings. Third, the whole-population CKD model had a statistically significant Hosmer–Lemeshow test (P = 0.041), indicating some departure from perfect calibration. Because this test is sensitive in large samples and may detect minor, clinically negligible deviations (22), the finding should be interpreted cautiously rather than as definitive evidence of clinically important miscalibration. Finally, the registry did not capture medication dosages, adherence, CKD stage, dialysis status, or longitudinal outcomes. Consequently, dose–response relationships and the prognostic significance of the sex-associated clinical characteristics identified in this study could not be assessed.

## Data Availability

The original contributions presented in the study are included in the article/Supplementary material, further inquiries can be directed to the corresponding author.
